# Home Labour Induction with Retrievable Prostaglandin Pessary and Continuous Telemetric Trans-Abdominal Fetal ECG Monitoring

**DOI:** 10.1371/journal.pone.0028129

**Published:** 2011-11-28

**Authors:** Zubair Rauf, Ediri O'Brien, Tamara Stampalija, Florin P. Ilioniu, Tina Lavender, Zarko Alfirevic

**Affiliations:** 1 Department of Women's and Children's Health, University of Liverpool, Liverpool Women's Hospital, Liverpool, United Kingdom; 2 School of Nursing, Midwifery & Social Work, University of Manchester, Manchester, United Kingdom; Institute of Clinical Effectiveness and Health Policy, Argentina

## Abstract

**Objective:**

To evaluate the feasibility of continuous telemetric trans-abdominal fetal electrocardiogram (a-fECG) in women undergoing labour induction at home.

**Study Design:**

Low risk women with singleton term pregnancy undergoing labour induction with retrievable, slow-release dinoprostone pessaries (n = 70) were allowed home for up to 24 hours, while a-fECG and uterine activity were monitored in hospital via wireless technology. Semi-structured diaries were analysed using a combined descriptive and interpretive approach.

**Results:**

62/70 women (89%) had successful home monitoring; 8 women (11%) were recalled because of signal loss. Home monitoring lasted between 2–22 hours (median 10 hours). Good quality signal was achieved most of the time (86%, SD 10%). 3 women were recalled back to hospital for suspicious a-fECG. In 2 cases suspicious a-fECG persisted, requiring Caesarean section after recall to hospital. 48/51 women who returned the diary coped well (94%); 46/51 were satisfied with home monitoring (90%).

**Conclusions:**

Continuous telemetric trans-abdominal fetal ECG monitoring of ambulatory women undergoing labour induction is feasible and acceptable to women.

## Introduction

The number of induced labours continues to increase with emerging evidence that induction of labour at term does not appear to increase caesarean delivery rates, but may benefit both mother and baby [1]–[4].

Home induction has emerged as an increasingly popular alternative to labour induction in hospital settings [5]–[6]. Unfortunately, safer alternatives to prostaglandins do not appear to be effective [7]–[8], and concerns for the fetal wellbeing during home inductions remain the key issue.

Induction of labour is also associated with decreased maternal satisfaction when compared with spontaneous labour [9]. Key negative influencing factors are the lack of comfort, privacy and flexibility in a hospital environment. Outpatient induction has been shown to increase maternal satisfaction; the home environment is private and familiar, thus increases control [10].

**Figure 1:**
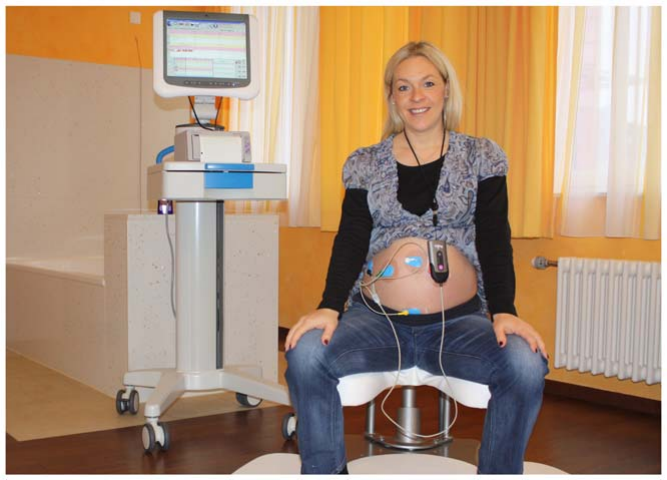
Fetal ECG monitoring device (MONICA AN24). Copyright and courtesy of Monica healthcare limited. Portable trans-abdominal fetal ECG monitoring device with 5 electrodes attached to the maternal abdomen.

**Figure 2:**
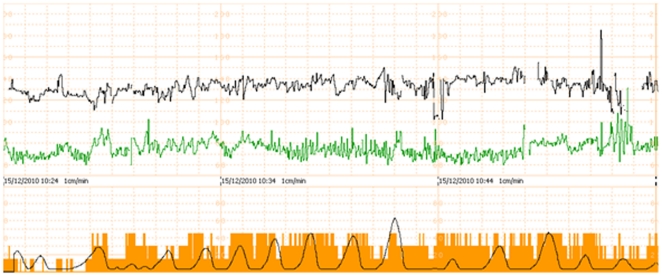
Monitoring display. Continuous monitoring display from the MONICA AN24 device of fetal and maternal heart rate, uterine contractions and maternal movements. (Fetal heart rate – black line (top); maternal heart rate – green line (middle); Uterine contractions – black line (bottom); maternal movements – orange bars).

A home induction protocol that complements use of slow release, retrievable prostaglandin pessary, with an user friendly remote fetal monitoring system, may provide a solution to the current concerns. Monica AN24 (Monica Healthcare Ltd) is a commercially available, wireless fetal-maternal monitoring device that allows remote non-invasive trans-abdominal monitoring of fetal heart activity (electrocardiography, ECG), uterine electrical activity (electromyography, EMG) and maternal heart rate in real-time (Figure 1 & 2). Our aim was to explore whether such remote continuous trans-abdominal fetal ECG monitoring in women undergoing induction of labour with retrievable, slow release prostaglandin pessary is feasible and acceptable to women.

## Methods

### Ethics Statement

This was a prospective cohort study carried out at Liverpool Women's Hospital Trust between January 2009 and December 2010, for which the Ethics Committee (Cheshire Research Ethics Committee) approval was obtained (REC-08/H1017/194), and Trust Research and Development approval reference (LWH0764). Written informed consent was obtained from all the participants in the study. The consent was given by the participants for their information to be stored in the hospital database and used for research by the responsible individuals.

There were 3 phases to this study:

### i) In-patient phase

We recruited a total of 16 women in order to test the feasibility of the telemetric a-fECG monitoring device. The signal was transmitted through the a-fECG monitoring device to a standard hospital monitor (PC), via ordinary cell phone (Bluetooth technology) to an online data reception server, accessible to the hospital staff through the internet. Simultaneous recordings of fetal ECG, uterine electrical activity and maternal heart rate were displayed with a delay of only 40 seconds.

All 16 women with singleton, cephalic pregnancies had their labour induced with slow release retrievable 10 mg dinoprostone pessary (Propess, Ferring Pharmaceuticals). The indications were: post-term (n = 8), gestational diabetes (n = 1), polyhydramnios (n = 2), proteinuria (n = 1), hypertension (n = 1), severe symphysis pubic dysfunction (n = 1), asthma (n = 1) and prolonged rupture of membranes (n = 1).

After obtaining written informed consent, the a-fECG monitoring device was attached. Concurrently, standard CTG Doppler was positioned over the a-fECG monitoring device and 30 minutes of simultaneous monitoring carried out. Fetal well-being was further confirmed for 60 minutes after insertion of vaginal dinoprostone. After this, standard CTG Doppler was removed whilst monitoring continued only with a-fECG monitoring device for a maximum of 24 hours or until delivery. Total duration for fetal ECG monitoring ranged between 3 and 20 hours.

### ii) Outpatient monitoring in healthy controls

In this phase, the a-fECG was used in 11 healthy pregnant volunteers at term. They all had low risk singleton, cephalic pregnancy. They were awaiting either spontaneous onset of labour or post-term induction of labour. These women were monitored whilst at home for up to 24 hours. Simultaneous recordings of fetal ECG, uterine activity and maternal heart were checked remotely for the signal quality. The duration of recordings ranged from 8 to 21 hours. The real time recordings could not be initiated despite electrode adjustments in 3 out of 11 women. Adequate signals were eventually achieved when devices were replaced. In one case there was a significant loss of signal at home which prompted us to amend the monitoring protocol. We included an option of advising subsequent participants that they may be asked to lie down for 30–60 minutes at home in order to reduce the signal loss due to excess mobility.

The quality of signal was quantified as the percentage of monitoring time during when it was possible to calculate the “two seconds average”. For each woman, the total a-fECG recording was divided into 2 second epochs. Each epoch was checked for a minimum of two consecutive fetal and maternal ECG complexes which, if present, enabled a heart rate to be calculated for that epoch. Success rate was expressed as a % of the number of epochs in which a 2 second averaged heart rate was derived, divided by the total number of epochs per hour.

We have pre-specified the targets for success rate based on the low clinical risk status of participant and expected significant mobility during day time i.e. >70% during day time and >80% during night time. As the overall percentage of success rate exceeded pre-specified criteria in 10 out of 11 participants, the quality of the monitoring was judged to be of sufficient quality to be tested for induction of labour at home.

We noted that one woman reported mild itching on the abdomen after removal of the abdominal electrode.

### iii) Home induction cohort

Women with healthy singleton pregnancy, cephalic presentation, gestational age ≥37^+0^ weeks and parity <4, who were scheduled for induction of labour were provided with information leaflets about the study and asked to sign a written consent. Only women with intact membranes, Bishop score <6; and normal trans-abdominal fetal ECG monitoring for 60 minutes after insertion of slow release retrievable dinoprostone pessary were eligible for home induction. We excluded women who did not have a birthing partner at home, did not have access to both telephone and transport, or were living > 60 minutes drive from hospital. Women with medical problems, previous caesarean delivery, maternal age < 18 years and contra-indication to slow release 10 mg dinoprostone pessary were also excluded.

All eligible women were sent home with 10 mg dinoprostone pessary while being continuously monitored telemetrically with trans-abdominal fetal ECG monitoring device.

Fetal ECG, uterine activity and maternal heart rate were reviewed at least once every hour by the midwifery staff. Women were advised to return to hospital if pessary fell out, in case of vaginal bleeding or ruptured membranes, painful uterine contractions requiring analgesia, continuous abdominal pain, or contractions occurring at a frequency of ≥3 in 10 minutes or lasting >60 seconds (allowing hospital delivery for all women). Women were also contacted in case of signal loss, trace concerns or transmission failure and were asked to return to hospital if connection could not be re-established.

If active phase of labour started within the battery life time (20–24 hours), the trans-abdominal fetal ECG monitoring was continued. Standard Doppler fetal heart rate (FHR) monitoring was used instead when batteries were used up, or when access to online data reception server was not available in the allocated delivery suite room. Standard Doppler FHR monitoring was also routinely used in the second stage of labour, because of the possibility of losing a-fECG signal secondary to fetal descent.

### iv) Qualitative assessment

Maternal views were assessed using semi-structured diaries. The diaries recorded women's ratings, on a 4 point scale, of how well they were coping, their comfort and satisfaction with outpatient experience. Additional free-text space allowed for comments on any side effects, concerns and positive or negative aspects of the care. Women were asked to complete diaries at least once every two hours during their time at home and indicate their location preference on each entry. Diaries were collected on admission to hospital and forwarded to the qualitative team. Mean scores were calculated for women's ratings of coping, comfort, satisfaction and location preference. An interpretive approach was utilised for all open responses [11]. Comments made in the free-text spaces of diaries were identified and summarised in order to contextualise women's ratings of their experience.

## Results

104 women consented to participate, of whom 24 laboured before the induction date. In addition, three women had Bishop Score >6 on the day of induction and there was one case of a cord presentation. Two women changed their mind and withdrew consent. In four instances it was not possible to establish the link between the a-fECG device and the online server (data reception point) despite adequate a-fECG recordings.

In total, 70 women fulfilled all eligibility criteria and were sent home with slow release dinoprostone pessary in situ (Table 1). Two women required caesarean section with suspicious FHR trace before active phase of labour was established (see below) and two women had Caesarean section for failed induction.

**Table 1:** Demographic and clinical characteristics of women who were monitored at home (n = 70).

Indication for induction	
*Post-term*	69
*Symphysis pubis dysfunction*	1
Gestational age (days)^a^	291 ( 2.5)
Body Mass Index (BMI) >35	6 (9%)
Nulliparous	45 (64%)
Bishop Score at start^b^	3 (0–5)
Successful induction (Bishop ≥6 within 24 hours)	53 (76%)
Additional dinoprostone	13 (19%)
Caesarean section	14 (20%)
*Pre-labour (suboptimal CTG)*	2
*Pre-labour (failed induction)*	2
*Intrapartum*	10
Birth weight (grams)^a^	3700 (396)
Apgar Score < 7 at 5 minutes^c^	3 (4 %)

a Mean (standard deviation).

b Median (range).

c Cord pHs - normal for these 3 cases.

### i) Home monitoring

Out of 70 women who were sent home, 62 (89%) were successfully monitored at home (Table 2). Three women (4%) developed non-reassuring FHR (suspicious) at home. They were instructed via telephone to remove dinoprostone pessary immediately and return back to hospital. In two cases, FHR became pathological requiring Caesarean section 2 and 4 hours later, respectively. Both babies were born with normal Apgar scores and normal arterial cord pH values of 7.37; BE −3 and 7.30; BE 2.3 respectively. In both cases no obvious cause for deteriorating a-fECG was identified at the time of caesarean delivery. In the third case a-fECG trace improved spontaneously to normal and remained normal until normal vaginal birth, 8 hours after return to hospital.

**Table 2:** Outcome of home monitoring.

Total number of women recruited	104	
Number of women withdrawn	34	
Number of women monitored at home	70	
Successful home monitoring	62 (89%)	
*<5 hours*	*11 (18%)*	
*5*–*10 hours*	*18 (29%)*	
*>10 hours*	*33 (53%)*	
Total monitoring time at home per woman a	10 h 35 min (1 h 55 min–22 h 4 min)	
Monitoring success rate per woman b	86% (10.5)	

Unsuccessful signal transmission at home		8 (11%)
*Total monitoring time in hours (median, range)*		*2 h 7 min (1 h 45 min* –*12 h)*

a Median (range).

b Overall monitoring success rate - mean (SD).

There were 2 cases of mild hyperstimulation at home. In both cases the midwife reviewing the a-fECG trace, noticed five or more contractions in 10 minutes lasting for over 30 minutes with completely reassuring FHR trace. Both women were contacted at home to inquire about the frequency and strength of contractions. One of these women confirmed feeling 1–3 contractions every 10 minutes which were of variable strength, but wished to stay at home as was coping quite well. Her trace was reviewed regularly telemetrically, and was found to be reassuring without further hyperstimulation. The second patient was already getting ready to make her way to the hospital. She confirmed to have ruptured her membranes at home and was feeling 4–6 painful uterine contractions every 10 minutes in the previous hour and a half. She arrived in hospital safely. Her first dinoprostone pessary had fallen out when her membranes ruptured and her contractions eased off completely within 3 hours of arrival. The decision was made to put in a 2nd dinoprostone pessary which was removed 4 hours later because of hyperstimulation without FHR changes. Terbutaline was given and vaginal delivery was achieved 4 hours later. Her baby was born with Apgar score of 6 at 5 minutes, but normal venous cord PH of 7.42 with BE of −4.4. Arterial cord sample was inadequate to be processed.

There were further 2 cases where the Apgar scores were <7 at 5 minutes. Both of these women were in hospital during active phase of labour. One baby had a rotational forceps delivery in theatre (cord arterial PH of 7.16 & BE −7.8). The second was a normal vaginal delivery after syntocinon infusion for 8 hours (cord arterial pH of 7.27, BE −5.2).

All the remaining 55 women who were successfully monitored at home delivered babies with Apgar scores of more than 7 at 5 minutes. Cord PH was successfully obtained in 43 of these 55 patients and there was no evidence of hypoxia or acidosis.

The duration of a-fECG monitoring at home ranged from 1 hour and 55 minutes to 22 hours and 4 minutes (median time 10 h 35 min). On average, the a-fECG was able to calculate and display recordings successfully in 86% of total recording time per woman (SD 10.5 %).

Prolonged signal transmission disruptions were encountered in 8 cases (11%) requiring recall to hospital for in-patient monitoring. None of these women were in established labour; they returned to hospital within 60 minutes once recalled. One out of 70 participants developed contact dermatitis at electrode site (found after delivery once the electrodes were removed). This resolved after local steroid ointment was applied for 1 week.

### ii) Women's views

Fifty-one completed diaries were returned, with a median of 6 entries per diary (range 1 to 10). Most women reported that they coped ‘well’ (n = 19) or ‘very well’ (n = 29) at home. Direct quotations are used to illustrate findings.

Comments indicated that the freedom to mobilise and familiar surroundings influenced their ability to cope with the irregular contractions.

*“Having irregular contractions. Can walk around house, have a drink, food, lie in bed etc”* [Participant Diary 33]

Women who coped ‘less well’ (n = 3) at home reported issues with device error (battery failure or signal loss), or anxiety from not receiving health professional feedback on progress of labour initiation.

*“Coping with the fact that I'm at home but feeling anxious that in eleven hours nothing has happened and I don't know if I'm any closer”* [Participant Diary 38]

The majority of women reported feeling ‘comfortable’ (n = 26), or very comfortable (n = 20) wearing the device, although a few were initially cautious about disturbing the pads/electrodes. Satisfaction ratings indicated that the majority of women were ‘satisfied’ (n = 21), or ‘very satisfied’ (n = 25) that they and their babies were being adequately monitored at home. Additional comments indicated that women's level of satisfaction was influenced by contact with the hospital; satisfaction levels increased following telephone contact from the hospital.

*“Received a phone call so know that I was being monitored”* [Participant Diary 22]

Location preferences expressed at each diary entry point demonstrated that women would rather be at home (n = 47) than in hospital (n = 4). Few women (n = 8) reported experiencing any side effects; minor discomfort from the pesssary (n = 4), nausea (n = 2), dizziness (n = 1) and headaches (n = 1) were highlighted. Twenty two women expressed feeling concerned at specific time points, usually when they felt they were no longer able to cope with contractions and labour pains, or when issues developed with the device.

*“Unsure if anything is working. Lots of backache – unsure if its contractions”* [Participant Diary 12]

Over 100 positive comments were made about monitoring at home. Freedom and comfort associated with being in their own surroundings and being able to sleep in their own beds increased their ability to relax. Women also appreciated the family support available to them at home.

*“The home monitoring did allow a lot more freedom and I believe that helped me to remain calm and ultimately helped with a straight forward labour….I also felt mobile and non-restricted whilst I was wearing the device”* [Participant Diary 42]

Overall, women felt that there were no negative aspects of being at home, although some comments (n = 34) were made revealing women's occasional worries, such as ‘*Am I being monitored?’; ‘Is everything OK?’* and the lack of direct feedback.

## Discussion

A combination of labour induction with slow release retrievable dinoprostone pessary with a portable home monitoring system seems a feasible alternative to in-patient induction for selected patients. There was a high acceptance rate of the overall package. Perceived benefits of being at home included freedom, mobility, privacy and comfort of home environment. Having family support was also beneficial. These findings resonate with existing literature [1]. Having contacts with health professionals, albeit by telephone, was welcomed; those who had less contacts reported more anxiety. The device was considered comfortable and non-restrictive; however, technical difficulties with monitoring increased the feelings of anxiety.

Technical difficulties with transmission were mainly associated with the online server failure i.e. a crash of the server's hard drive needed to receive and store FHR data. Inability to telemetrically monitor the FHR traces in these cases necessitated recall of women to the hospital.

In the pilot phases skin needed to be exfoliated with medical sand paper so the electrodes could pass the impedance test. In the main study this was only done in women who had used moisturizing gels/lotions on the abdomen; there was no need to prepare the skin vigorously in the rest of the women. Therefore, in its current format, this protocol may be suitable for units who have the resources to train dedicated staff to provide this service. The process of electrode applications and mobile technology set up would have to be less challenging for routine use by busy clinicians.

Clearly, this study was not large enough to address the issue of safety of outpatient induction with prostaglandins. There are clinicians who will never approve of this concept claiming that the risk of hyperstimulation and possible fetal distress is simply too great. Our dinporostone associated hyperstimulation rate experienced during home monitoring (3.2%) was consistent with the meta-analyses by Austin et al. [12] and Kelly et al. [13] reporting the rates of 6.3% and 7.5%, respectively. In addition, we had two cases of unexplained, persistent FHR abnormalities. Given that both babies were born in excellent condition, it is possible that these changes would have resolved spontaneously.

Ideally, a randomised controlled trial would have to be carried to confirm that outpatient induction with (or without) continuous fetal monitoring is as safe as hospital induction. Women need to be able to return to hospital quite quickly i.e. easy access to transport is essential. Assuming that the induction agent would be the same in both settings (e.g. slow release dinoprostone), the expected incidence of clinically relevant adverse neonatal outcome should not exceed 1%. If one assumes that an additional risk of adverse outcome from outpatient induction of around 0.2% (1 in 500 home inductions) would be totally unacceptable, nearly 90,000 women would have to be randomised. Such a study would be needed to exclude the possibility that the risk of outpatient induction is not 20% greater compared with hospital induction (1.2% vs. 1%). Less satisfactory, but more practical alternative is to encourage units who practice outpatient induction to keep reporting not only successes, but also failures and any adverse outcomes in an unbiased fashion.

Our pilot study confirms that the use of telemetric trans-abdominal fECG monitoring device in ambulatory women undergoing labour induction is feasible and acceptable to women, resulting in high level of satisfaction with the received care. The quality of remote signal allowed clinical decision making in real time in all women. The trade-off between women's choice, safety issues and cost-effectiveness will be the main issue once the technology becomes sufficiently robust and even more user friendly.
